# Dynamic Distance Measurement Based on a Fast Frequency-Swept Interferometry

**DOI:** 10.3390/s22134771

**Published:** 2022-06-24

**Authors:** Yuru Chen, Xiaohua Lei, Lin Xiao, Peng Zhang, Xianming Liu

**Affiliations:** The Key Lab of Optoelectronic Technology & Systems Ministry of Education, College of Optoelectronic Engineering, Chongqing University, Chongqing 400044, China; chenyuru@cqu.edu.cn (Y.C.); linxiao@cqu.edu.cn (L.X.); zhangpeng@cqu.edu.cn (P.Z.); xianming65@163.com (X.L.)

**Keywords:** frequency-sweeping interferometry, dynamic distance measurement, Doppler-induced error compensation

## Abstract

To improve the precision of dynamic distance measurement based on the frequency-swept interferometry (FSI) system, a Doppler-induced error compensation model based on a scheme increasing the frequency sweeping rate is proposed. A distance demodulation method based on a Fourier transformation is investigated when the defined quasi-stationary coefficient approaches a constant. Simulations and experiments based on dynamic distance with a sinusoidal change demonstrate that the proposed method has a standard deviation of 0.09 μm within a distance range of 4 μm at a sweeping rate of 60 KHz.

## 1. Introduction

Frequency-swept interferometry (FSI) technology has many advantages, such as high precision, high response speed, etc., when applied in absolute distance measurement for a static target. However, it will introduce the Doppler-induced error for a dynamic target [1,2,3]. The common way to solve this problem is to introduce additional hardware or components to increase a known quantity. For example, Richard Schneider et al. [4] devised a dual-laser sweeping system. The Doppler-induced error is eliminated by averaging the two-phase shifts produced by the frequency-sweeping of two lasers in opposite directions. Warden et al. [5,6] presented a dual-FSI system with a gas absorption cell to achieve dynamic OPD measurement at any sampling point. Pollinger and Liu et al. [7,8] used an additional heterodyne interferometry to directly measure the target movement and used an FSI system to calculate the distance. Shao Bin et al. [9] reported a fixed-frequency laser to measure the target velocity and used a FSI system to calculate the distance. Although these methods all successfully reduced the Doppler-induced error, they also increased the complexity, both for the demodulation algorithm and the measurement system.

To simplify hardware configuration, based on the hypothesis that the target is drifting at a constant speed or acceleration during the laser sweeping, Swinkels et al. [10] proposed an algorithm to combine four consecutive phase measurements instead of the normal two to reduce the Doppler-induced error, without utilizing auxiliary laser. Z. Liu et al. [2,11] proposed real-time models using the Kalman filter with one frequency-sweeping laser and in-phase and quadrature detection, which also assumed the target varied by a constant velocity or acceleration during the laser sweeps.

Aiming at measuring the dynamic distance where the target velocity is an arbitrary variable, in this paper, a Doppler-induced error compensation model based on a scheme increasing the frequency sweeping rate is proposed with only one frequency-sweeping laser. A distance demodulation method combining a Fourier transformation with a correlation-like algorithm is investigated when the defined quasi-stationary coefficient approaches a constant. Without utilizing auxiliary laser and complex algorithms, this method can greatly simplify the distance measurement based on the fast FSI.

## 2. Principle of Reducing the Doppler-Induced Error

**Figure 1:**
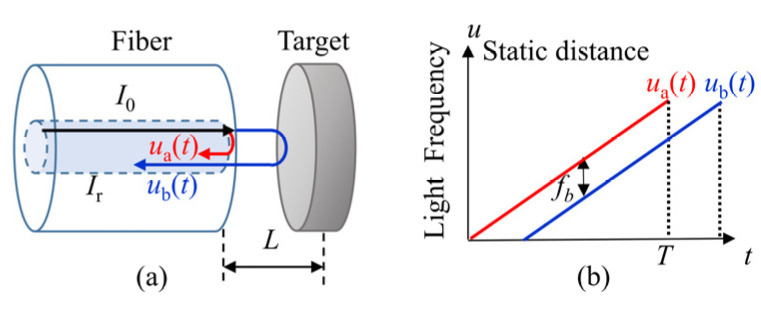
Model of the static distance. (**a**) The interference model of the static distance; (**b**) the light frequency change in a sweeping circle.

Figure 1a shows the schematic of a static distance measurement with an FSI system. One part of the light beam is reflected at the end face of the fiber, its frequency is recorded as *u_a_*(*t*). The rest of light is transmitted out of the fiber and reflected by the target, then coupled back into the fiber, its frequency is recorded as *u_b_*(*t*). The beat frequency can be written as [12]:

(1) $$\begin{array}{ccc} f_{b} & = & u_{a}\left(t\right)-u_{b}\left(t\right)=\frac{\Delta u}{T}\cdot \frac{2nL_{sta}}{c},\;\;\;\;\;0\leq t\leq T\\ u\left(t\right) & = & u\left(0\right)+\frac{\Delta u}{T}t \end{array}$$

where *n* is the refractive index between the fiber end-face and the target, *n* = 1 when in the air; *u* is the optical frequency; Δ*u* is the optical frequency range; *c* is the speed of light; *f_b_* is the beat frequency of the FSI signal; *t* is the sweeping time; and *T* is the sweeping cycle.

Specifically, the light frequency *u_a_*(*t*) and *u_b_*(*t*) vary linearly in a sweeping cycle and the time delay between two beams is constant, as shown in Figure 1b. So, the beat frequency *f_b_* is a constant in a sweeping cycle. According to Equation (1), the static distance is:

(2) $$L_{sta}=\frac{cT}{2\Delta u}f_{b}$$

**Figure 2:**
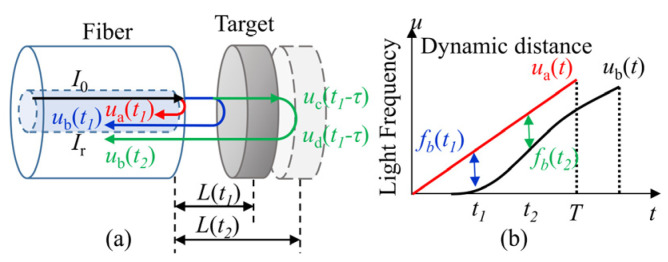
Model of the dynamic distance. (**a**) The interference model of the dynamic distance, a and b are the position out and in the fiber end-face, c and d are the position at the target, *t*_1_ and *t*_2_ are the different moments when the target is moving; (**b**) the beat frequency of the dynamic distance during one sweeping cycle.

However, for a dynamic target shown in Figure 2, the distance *L* changes during a sweeping cycle, so the beat frequency *f_b_* is no longer a constant. To analysis the instantaneous beat frequency, the light frequency reflected at fiber end-face is still assumed as *u_a_*(*t*), the light frequency arriving at the target is *u_c_*(*t*), the light travelling time from the fiber end-face to the target is τ, then, *u_c_*(*t*) can be written as:

(3) $$\begin{array}{ll} u_{c}\left(t\right) & =u_{a}\left(t-\tau \right),\;0\leq t\leq T\\ \tau & =\frac{L\left(t\right)}{c} \end{array}$$

Consider the Doppler effect, the light frequency reflected at the target *u*_d_(*t*) is [13]:

(4) $$u_{d}\left(t\right)=u_{c}\left(t\right)\frac{c-v\left(t\right)}{c+v\left(t\right)}\approx u_{c}\left(t\right)\left[1-\frac{2v\left(t\right)}{c}\right],\;0\leq t\leq T$$

where *v*(*t*) is the velocity of the target.

Assume that the light traveling time from the fiber end-face to the target is equal to the time from the target to the fiber end-face, the light frequency going back to the fiber end-face *u_b_*(*t*) is:

(5) $$u_{b}\left(t\right)=u_{d}\left(t-\tau \right),\;0\leq t\leq T$$

Then the instantaneous beat frequency is:

(6) $$\begin{array}{ll} f_{bd}\left(t\right) & =u_{a}\left(t\right)-u_{b}\left(t\right)=u_{a}\left(t\right)-u_{a}\left(t-2\tau \right)\left[1-\frac{2v\left(t-2\tau \right)}{c}\right]\\ & \approx u_{a}\left(t\right)\frac{2v\left(t\right)}{c}+2\frac{\Delta u}{T}\tau ,\;0\leq t\leq T \end{array}$$

According to Equation (2), the dynamic instantaneous distance in a sweeping cycle is calculated to be:

(7) $$\begin{array}{ll} L_{dyn}\left(t\right) & =\frac{cT}{2\Delta u}\left[u_{a}\left(t\right)\frac{2v\left(t\right)}{c}+2\frac{\Delta u}{T}\tau \right]=L\left(t\right)+u_{a}\left(t\right)\frac{T}{\Delta u}v\left(t\right)\\ & =L\left(t\right)+\frac{u_{a}\left(t\right)}{\Delta u\cdot f_{sweep}}v\left(t\right)=L+L_{doppler},\;0\leq t\leq T \end{array}$$

where *f_sweep_* = 1/*T*, *f_sweep_* is the sweeping rate.

**Figure 3:**
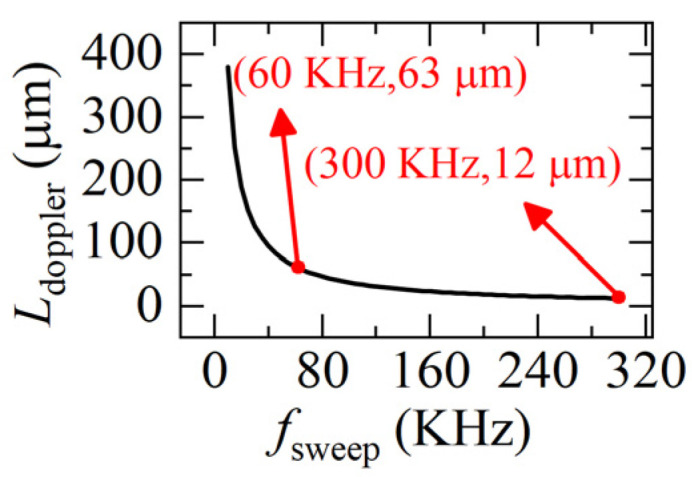
The relationship between the sweeping rate *f_sweep_* and the Doppler-induced error *L_error_*. (Δ*u* = 5.1 × 10^12^ Hz, *u_a_* = 1.9365 × 10^14^ Hz, *v* = 0.1 m/s, *a* = 0.1 m/s^2^).

The first term in Equation (7) is the real distance *L*, the second term is the Doppler-induced error *L_error_*. We can see, *L_error_* is related to the velocity of the target *v*(*t*) and the sweeping rate of the light *f_sweep_*. As *f_sweep_* increases, *L_error_* rapidly decreases and then tends to be constant. When *v*(*t*) and light frequency are fixed, the greater the *f_sweep_* is, the smaller the *L_doppler_* is, as shown in Figure 3.

Although increasing the sweeping rate helps to reduce the Doppler-induced error *L_doppler_*, it is still 12 μm at a very high sweeping rate (300 KHz), which is too large when compared to the distance variation of 0.3 μm.

The real instantaneous distance, which is the first term in Equation (7), can be further described to be [2]:

(8) $$L\left(t\right)=L\left(0\right)+\int_{0}^{t}v\left(t\right)dt=L\left(0\right)+v\left(0\right)t+\frac{1}{2}at^{2},\;0\leq t\leq T$$

where *L*(0) is the initial distance in one sweeping cycle and *a* is the acceleration.

From Equation (7), we can see that when the sweeping cycle becomes small, the distance change in one sweeping cycle will also be small. At this time, take Equation (8) into Equation (7), let *L_dyn_*(*T*), which is the distance at the end moment T of a sweeping cycle, represent the distance after the whole sweeping cycle. It is:

(9) $$\begin{array}{ll} L_{dyn}\left(T\right) & =L\left(0\right)+v\left(0\right)T+\frac{1}{2}aT^{2}+\left(v\left(0\right)+aT\right)\frac{Tu_{a}\left(T\right)}{\Delta u}\\ & =L\left(0\right)+v\left(0\right)T\left(1+\frac{u_{a}\left(T\right)}{\Delta u}\right)+\left(\frac{1}{2}+\frac{u_{a}\left(T\right)}{\Delta u}\right)aT^{2} \end{array}$$

In Equation (9), the sweeping cycle *T* is assumed to be sufficiently small. In this case, the distance change in one cycle is considered to be very small, the velocity or acceleration is regarded as a constant.

Define a Doppler coefficient to be:

(10) $$A_{doppler}=1+\frac{u_{a}\left(T\right)}{\Delta u}$$

Let both sides of Equation (9) be divided by Equation (10) after moving *L*(0) in Equation (9) from right to the left. The distance variation is:

(11) $$\Delta L_{mear}\left(T\right)=\frac{L_{dyn}\left(T\right)-L\left(0\right)}{A_{doppler}}=v\left(0\right)T+\frac{\Delta u+2u_{a}\left(T\right)}{2\left(\Delta u+u_{a}\left(T\right)\right)}aT^{2}$$

Then after further suppressing the Doppler-induced error, the measured distance is:

(12) $$\begin{array}{ll} L_{mear}\left(T\right) & =L\left(0\right)+\Delta L_{mear}\left(T\right)=L\left(0\right)+v\left(0\right)T+\frac{\Delta u+2u_{a}\left(T\right)}{2\left(\Delta u+u_{a}\left(T\right)\right)}aT^{2}\\ & =L\left(0\right)+v\left(0\right)\frac{1}{f_{sweep}}+\frac{\Delta u+2u_{a}\left(T\right)}{2\left(\Delta u+u_{a}\left(T\right)\right)}\cdot \frac{a}{f_{sweep}^{2}} \end{array}$$

The principle residual error is expressed as:

(13) $$L_{error}\left(T\right)=L\left(T\right)-L_{mear}\left(T\right)=\frac{u_{a}\left(T\right)}{2\left(\Delta u+u_{a}\left(T\right)\right)}aT^{2}=\frac{u_{a}\left(T\right)}{2\left(\Delta u+u_{a}\left(T\right)\right)}\cdot \frac{a}{f_{sweep}^{2}}$$

**Figure 4:**
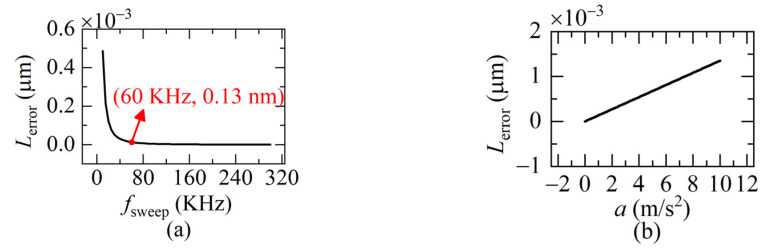
The residual error. (**a**) *L_error_* versus sweeping rate; (**b**) *L_error_* versus the acceleration *a* (Δ*u* = 5.1 × 10^12^ Hz, *u_a_* = 1.9365 × 10^14^ Hz, *v* = 0.1 m/s, *a* = 0.1 m/s^2^, *f_sweep_* = 60 KHz).

From Equation (13) we can see that *L_error_* is related to *a* and *T*. When *T* is getting smaller, which means the sweeping rate *f_sweep_* is higher, *L_error_* decreases. The larger *a* is, the larger *L_error_* becomes. Further simulation based on Equation (13) is given in Figure 4. When *f_sweep_* is 60 KHz, the residual error *L_error_* has been reduced to 0.13 nm and *L_error_* is only 1.3 nm even when the acceleration *a* is 10 m/s^2^. That is, we can suppress the Doppler-induced error to the order of a nanometer, by increasing the sweeping rate of the light frequency to some extent.

## 3. Demodulation of Dynamic Distance

### 3.1. Applicable Conditions of Frequency Demodulation

For a static distance measurement, the interference signal in one sweeping cycle is [14]:

(14) $$s_{sta}\left(t\right)=\cos\left(2\pi f_{b}t+\varphi_{0}\right),\;0\leq t\leq T$$

**Figure 5:**
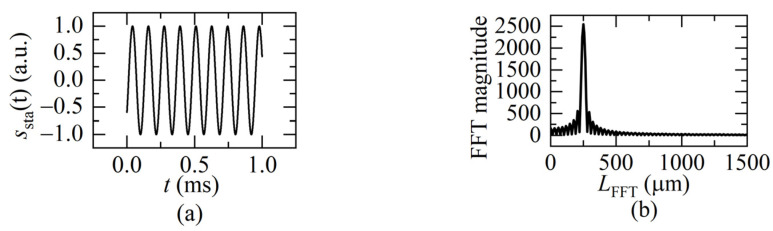
(**a**) The static FSI signal in one sweeping cycle (*L_sta_* = 250 μm); (**b**) the beat frequency result of the static FSI signal after the FFT.

It is a standard cosine function with a single beat frequency *f_b_*, as shown in Figure 5a. The fast Fourier transformation (FFT) can be used to get its interference frequency in the frequency domain shown in Figure 5b, then calculate the distance according to the relationship between beat frequency and distance using Equation (2).

For a dynamic target, the interference signal can be derived by the instantaneous beat frequency *f_bd_* in Equation (6) as:

(15) $$s_{dyn}(t)=\cos\left\{2\pi \int_{0}^{t}f_{bd}dt+\varphi_{0}\right\}=\cos\left\{2\pi \int_{0}^{t}\left[u_{a}(t)\frac{2v(t)}{c}+\frac{\Delta u}{T}\cdot \frac{2L(t)}{c}\right]dt+\varphi_{0}\right\},\;0\leq t\leq T$$

**Figure 6:**
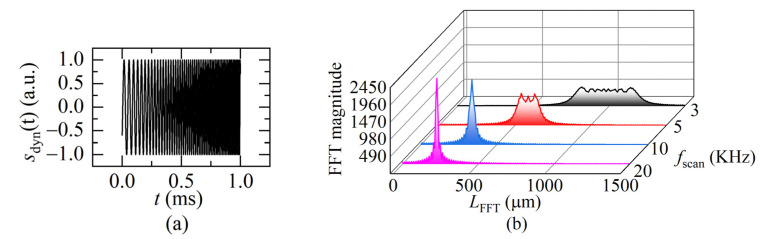
(**a**) The dynamic FSI signal (*L*(0) = 250 μm, *f_sweep_* = 1 KHz, *v* = 0.01 m/s, *a* = 50 m/s^2^) in one sweeping cycle; (**b**) the FFT transformation of the different sweeping rate. (*L*(0) = 250 μm, *a* = 200 m/s^2^, *v* = 0.1 m/s).

Equation (15) indicates that the beat frequency *f_bd_* varies as the sweeping time *t*. The interference signal is not a uniform distribution, as shown in Figure 5a. To understand the signal from the frequency domain, the instantaneous beat frequency *f_bd_* is obtained after applying the FFT to Equation (15), as shown in Figure 6. When *f_sweep_* is low, the FFT spectrum contains multiple instantaneous frequencies (black curve), which correspond to the instantaneous distance change because of the movement of the target in a sweeping cycle. When *f_sweep_* is gradually rising, the frequencies gradually merge (red curve).

When the ratio of *f_sweep_* to the movement frequency of the target reaches “a certain value”, the frequencies merge into one peak (blue curve). That means, there is only one beat frequency, which is the same as the static distance situation (shown in Figure 5b). It can be considered that the movement of the target compared to the light frequency sweeping is quasi-static. Therefore, the FFT frequency algorithm, instead of the complex demodulation methods [15,16,17,18], can be used to demodulate the beat frequency then calculate the distance at the end moment of one sweeping cycle using Equation (12).

To analyze the quantitative condition of the quasi-static state, define *ρ* as the quasi-stationary coefficient to describe the ratio of the static beat frequency to the dynamic beat frequency, which is:

(16) $$\rho =\frac{f_{b}}{f_{bd}}=\frac{2\frac{\Delta u}{T}\cdot \frac{L(t)}{c}}{\left(u_{a}(0)+\frac{\Delta u}{T}\cdot t\right)\cdot \frac{2v(t)}{c}+2\frac{\Delta u}{T}\cdot \frac{L(t)}{c}}\approx \frac{\Delta uL(T)}{u_{a}(T)v(T)\cdot \frac{1}{f_{sweep}}+\Delta uL(T)},\;(t\approx T)$$

Equation (16) shows that *ρ is* related to *v*(*t*) and *f_sweep_*. Suppose *v*(*t*) is a cosine function to imitate the target moving at an arbitrary speed. That is:

(17) $$v=\frac{dL}{dt}=2\pi \omega \cdot A\cos\left(2\pi \omega t\right),\;0\leq t\leq T$$

where *A* is the range of distance change and *ω* is the frequency.

**Figure 7:**
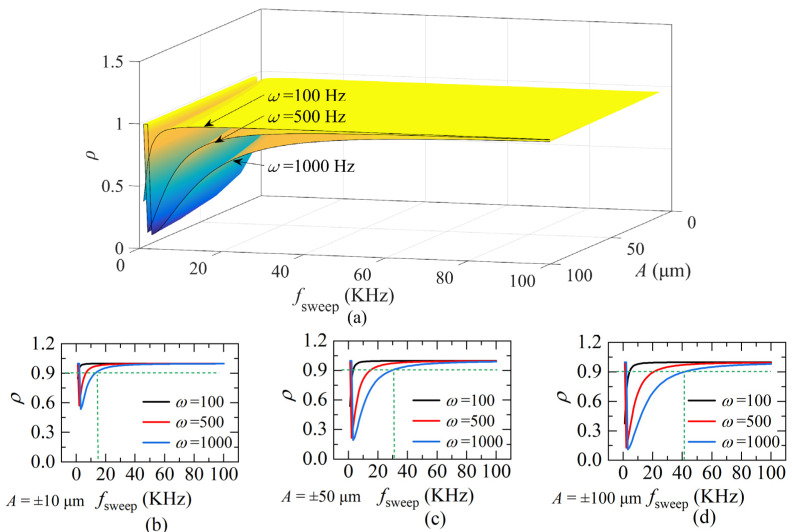
(**a**) The quasi-static coefficient *ρ* of different *f_sweep_*, the frequency *ω* of the distance and the amplitude *A*; (**b**) *ρ* when *A* = ±10 μm; (**c**) *ρ* when *A* = ±50 μm; (**d**) *ρ* when *A* = ±100 μm (*L*(0) = 800 μm, Δ*u* = 5.1 × 10^12^ Hz, *u_a_* = 1.9365 × 10^14^ Hz).

The value *ρ* is simulated according to Equations (16) and (17), and plotted in Figure 7. The value of *ρ* is related to *ω*, *A* and *f_sweep_*, as shown in Figure 7a. To understand the relationship between *ρ* and *f_sweep_* more clearly, extract some specific curves when *A* is ±10 μm, ±50 μm, and ±100 μm and plot in Figure 7b–d. It shows that, as the sweeping rate *f_sweep_* of the laser increases, *ρ* gradually approaches a constant, which indicates the movement of the target compared to the light frequency-sweeping is quasi-static. However, the quasi-static state is affected by *ω* and *A*. Comparing the *f_sweep_* corresponding to the points with *ρ* = 0.9 and *ω* = 1000 Hz in Figure 7b–d, it can be seen that *ρ* rises slower when *A* is larger. That is, the larger the *ω* or *A*, the longer *ρ* takes to reach a constant, the larger the corresponding *f_sweep_*. Therefore, before using the FFT frequency algorithm, the highest frequency *ω_max_* and the range of distance change *A* need to be calculated. For a simple calculation, when *f_sweep_* is around 20~50 times larger than the highest frequency of distance, the FFT algorithm would work, according to Figure 7 and is demonstrated by our practice.

### 3.2. Demodulation Algorithm

The FSI signal is actually a discrete sampled signal. The sampling points are equal to the swept points of the light frequency. To increase the resolution of FFT, a zero-padding extension to the discrete signal is often applied. Then, according to the FFT algorithm:

(18) $$S\left[k\right]=\sum_{n=0}^{N_{FFT}-1}s_{dyn}\left[n\right]e^{-i\frac{2\pi }{N_{FFT}}nk}.$$

where *s*[*n*] is the discrete form of Equation (15), *N_FFT_* is the length of *s*[*n*] after applying the zero-padding extension.

The distance is calculated to be:

(19) $$L_{FFT}=\frac{cT}{2\Delta u}\cdot \frac{n_{\max}}{N_{FFT}}$$

where *n*_max_ is the location of the peak frequency.

The demodulated distance *L_corr_* contains the real distance *L* and the Doppler-induced error *L_doppler_*. According to Equations (11) and (12), the measured distance *L_mear_* after suppressing the Doppler-induced error is:

(20) $$L_{mear}=\frac{L_{FFT}-L\left(0\right)}{A_{doppler}}+L\left(0\right)$$

**Figure 8:**
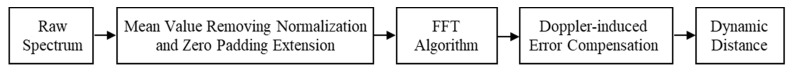
Data processing flow.

The data processing flow is shown in Figure 8. The first process of the raw spectrum is the mean value removing, normalization and zero-padding extension. Then, the distance is obtained using the *FFT* algorithm. After the Doppler-induced error compensation according to Equation (20), the dynamic distance can be demodulated.

### 3.3. Simulation

In order to verify the analysis above, a dynamic distance *L*(*t*) varies as a sinusoidal function which is given as:

(21) L(t)=L(0)+Asin(2πω⋅t),0≤t≤T

**Figure 9:**
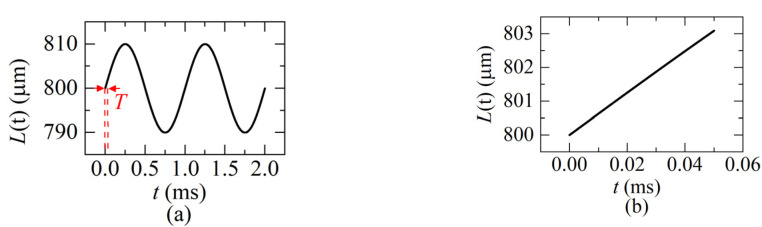
(**a**) A dynamic distance varies as a sinusoidal function (*L*(0) = 800 μm, *ω* = 1 KHz, *A* = ±10 μm, *v*_max_ = 0.06 m/s); (**b**) the distance in a sweeping cycle (*f_sweep_* = 20 KHz).

Figure 9a shows the distance *L*(*t*) varies as a sinusoidal function according to Equation (21). When the sweeping cycle *T* = 0.05 ms and the sweeping rate *f_sweep_* = 1/*T* = 20 KHz, the variation of the distance in one sweeping cycle is shown as Figure 9b, which is corresponding to the red part in Figure 9a.

**Figure 10:**
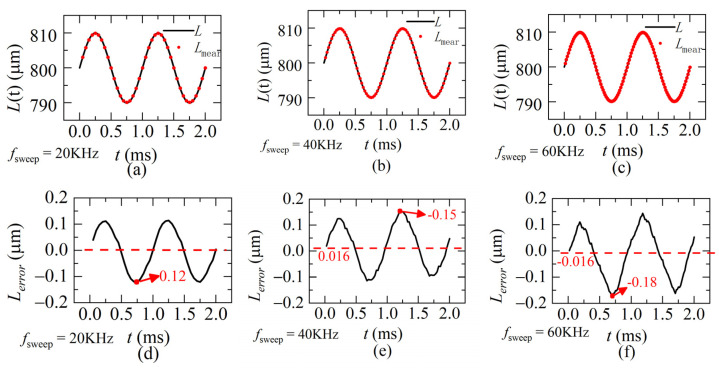
The simulation distances and the errors. *L* is the ideal distance, *L_mear_* is the demodulated distance, *L_error_* is the demodulation error between *L* and *L_mear_* (**a**,**d**) *f_sweep_* = 20 KHz (**b**,**e**) *f_sweep_* = 40 KHz (**c**,**f**) *f_sweep_* = 60 KHz (*L*(0) = 800 μm, ω = 1 KHz, A = ±10 μm).

According to the quasi-static state condition, when the frequency of the dynamic distance is 1 KHz, the sweeping rates of the laser *f_sweep_* is set 20 KHz, 40 KHz or 60 KHz. The demodulation results are calculated by our proposed method and shown in Figure 10a–c, respectively. The corresponding demodulation errors for each sweeping rate are shown in Figure 10d–f. The mean value of the absolute demodulation error is 0–0.019 μm and the maximum value is 0.13–0.16 μm, accounting for 0.3% of the demodulation range (60 μm). Results show our proposed method is able to achieve high demodulation accuracy even when the dynamic distance is in the form of a sinusoidal function, which means both velocity and acceleration are changing during sweeping.

## 4. Experiment

**Figure 11:**
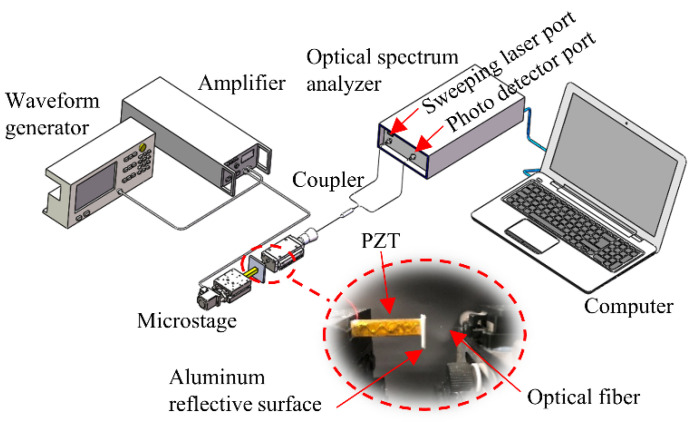
Experimental system diagram.

The dynamic distance measurement experiment was carried out to verify the proposed method, as shown in Figure 11. The distance was formed by the gap between the fiber end-face and the PZT (Pk4FA2H3P2, Thorlabs, Newton, NJ, USA). The PZT was driven by a signal generator (DG4102, RIGOL) and the amplifier (Has 4011, NF Corporation, Yokohama, Japan). The optical fiber was connected to a frequency-swept laser (Arcadia Optronix, Zhuhai, China, GC-760001c-01) and the photodetector module (Xilinx Artix-7, Conquer, Beijing, China). The entire setup was placed on a vibration-isolated optical stage.

The sinusoidal vibration frequency of the *PZT* was set to 100 Hz, 500 Hz, and 1 KHz, and the laser was scanned at different frequency intervals. The dynamic distance was expressed as:

(22) $$L=L\left(0\right)+A_{PZT}\sin(2\pi \omega_{PZT}\times t)$$

where *L*(0) is 250 μm, *ω_PZT_* is the PZT vibration frequency, and the vibration amplitude *A_PZT_* = ±2 μm.

**Figure 12:**
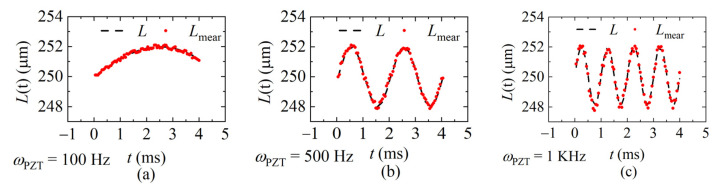
The demodulated distance at the sweeping rate of 20 KHz. (**a**) *ω_PZT_* = 100 Hz (*v*_max_ = 0.001 m/s); (**b**) *ω_PZT_* = 500 Hz (*v*_max_ = 0.006 m/s); (**c**) *ω_PZT_* = 1 KHz (*v*_max_ = 0.01 m/s).

**Figure 13:**
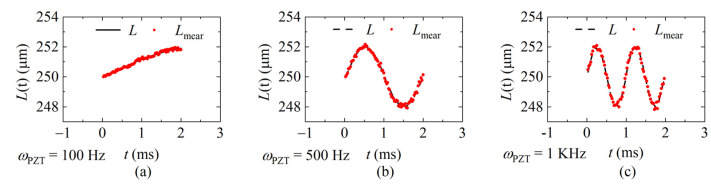
The demodulated distance at the sweeping rate of 40 KHz. (**a**) *ω_PZT_* = 100 Hz (*v*_max_ = 0.001 m/s); (**b**) *ω_PZT_* = 500 Hz (*v*_max_ = 0.006 m/s); (**c**) *ω_PZT_* = 1 KHz (*v*_max_ = 0.01 m/s).

**Figure 14:**
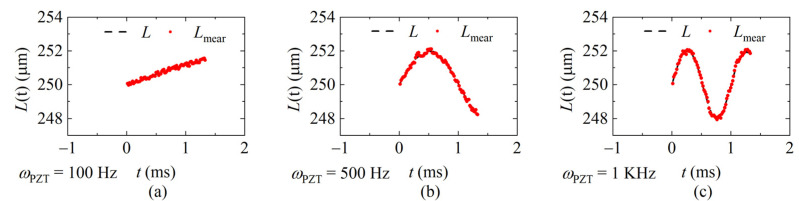
Measured values of different signal frequencies at 60 KHz sweeping rate. (**a**) *ω_PZT_* = 100 Hz (*v*_max_ = 0.001 m/s); (**b**) *ω_PZT_* = 500 Hz (*v*_max_ = 0.006 m/s); (**c**) *ω_PZT_* = 1 KHz (*v*_max_ = 0.01 m/s).

The interference spectra of each sweeping cycle were collected by the laser at the sweeping rates of 20 KHz, 40 KHz, and 60 KHz, respectively, and the demodulation results were calculated according to the data processing flow shown in Figure 8 and given in Figure 12, Figure 13 and Figure 14. Figure 12 shows the demodulation distances with *f_sweep_* = 20 KHz, and the frequency of the distance is *ω_PZT_* of 100 Hz, 500 Hz, and 1 KHz, respectively. Figure 13 shows *f_sweep_* = 40 KHz and Figure 14 shows *f_sweep_* = 60 KHz. The red dots indicate the demodulated distance, and the black delineated lines are the ideal distance according to the parameters of the signal generator.

**Figure 15:**
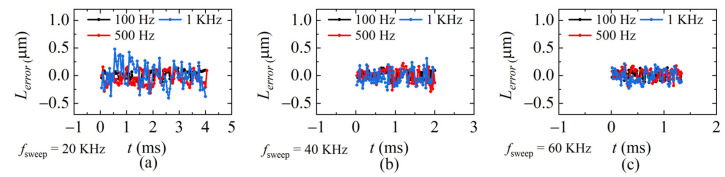
Errors at sweeping rates of (**a**) 20 KHz, (**b**) 40 KHz, and (**c**) 60 KHz, respectively.

**Figure 16:**
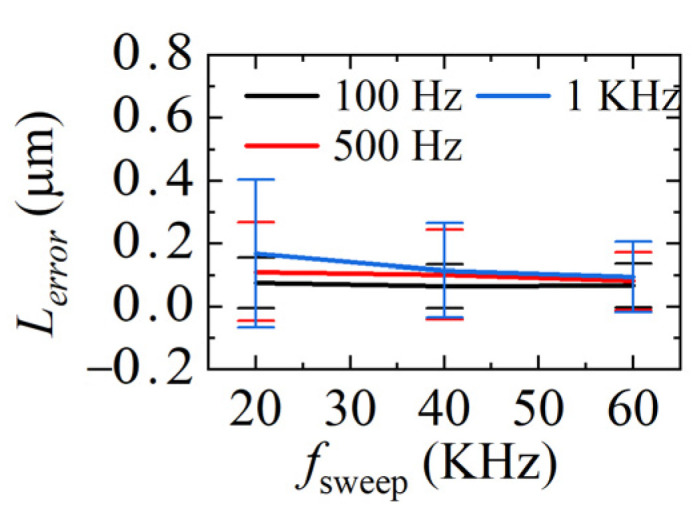
Comparison of mean values of gap measurement errors at different sweeping rates.

The demodulation errors between the demodulated distances and ideal distances in Figure 12, Figure 13 and Figure 14 are calculated and given in Figure 15. The average standard deviation is 0.14 μm, 0.11 μm, and 0.09 μm when *f_sweep_* is 20 KHz, 40 KHz, and 60 KHz, respectively. The larger *f_sweep_*, the smaller the fluctuation of the demodulated distance, which means the smaller the error, the mean value of the demodulation errors in each condition is plotted in Figure 16. It can be seen that when *f_sweep_* is fixed, *L_error_* increases with the increase in the *ω_PZT_*; when *ω_PZT_* is fixed, *L_error_* decreases with the increase in *f_sweep_*. The maximum error is 0.41 μm and the minimum error is 0.001 μm.

**Figure 17:**
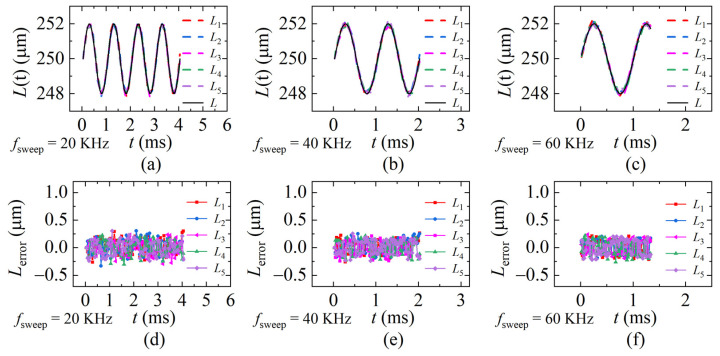
The distances and the errors of the repeatability experiments. *L* is the ideal distance, *L*_1_~*L*_5_ are the results of the five identical experiments (**a**,**d**) *f_sweep_* = 20 KHz; (**b**,**e**) *f_sweep_* = 40 KHz; (**c**,**f**) *f_sweep_* = 60 KHz (*ω_PZT_* = 1 KHz, *v*_max_ = 0.01 m/s).

To verify the repeatability of the proposed method, five identical dynamic experiments were conducted when *f_sweep_* is 20 KHz, 40 KHz, and 60 KHz. The PZT vibration-frequency *ω_PZT_* is 1 KHz in the repeatability experiments. The demodulation results were calculated and are shown in Figure 17a–c. Corresponding errors are shown in Figure 17d–f. The average standard deviation of each sweeping rate is 0.133 μm, 0.122 μm, and 0.123 μm. It can be seen from the comparison that the larger *f_sweep_* is, the smaller the error is when *ω_PZT_* is a constant.

## 5. Conclusions

A Doppler-induced error compensation model based on a scheme to increase the frequency sweeping rate is proposed. A distance demodulation method combining a Fourier transformation and a correlation-like algorithm is investigated when the defined quasi-stationary coefficient approaches a constant. Simulations and experiments based on dynamic distance with a sinusoidal change demonstrate that the proposed method has a standard deviation of 0.09 μm within a distance range of 4 μm at a sweeping rate of 60 KHz.

The proposed method is based only on the basic FSI system without any additional devices which can greatly simplify the hardware system; it is also based on the FFT and correlation-like algorithm instead of complex methods, which can simplify the calculation.

## Data Availability

The data that support the findings of this study are available from the corresponding author upon reasonable request.
